# On the impact of different approaches to classify age-related macular degeneration: Results from the German AugUR study

**DOI:** 10.1038/s41598-018-26629-5

**Published:** 2018-06-06

**Authors:** Caroline Brandl, Martina E. Zimmermann, Felix Günther, Teresa Barth, Matthias Olden, Sabine C. Schelter, Florian Kronenberg, Julika Loss, Helmut Küchenhoff, Horst Helbig, Bernhard H. F. Weber, Klaus J. Stark, Iris M. Heid

**Affiliations:** 1 Department of Genetic Epidemiology, University of Regensburg, Regensburg, Germany; 2 Department of Ophthalmology, University Hospital Regensburg, Regensburg, Germany; 3 Institute of Human Genetics, University of Regensburg, Regensburg, Germany; 4 Statistical Consulting Unit StaBLab, Department of Statistics, Ludwig-Maximilians-University, Munich, Germany; 5 Centre for Clinical Studies, University Hospital Regensburg, Regensburg, Germany; 6 Division of Genetic Epidemiology, Medical University of Innsbruck, Innsbruck, Austria; 7 Medical Sociology, Institute of Epidemiology and Preventive Medicine, University of Regensburg, Regensburg, Germany

## Abstract

While age-related macular degeneration (AMD) poses an important personal and public health burden, comparing epidemiological studies on AMD is hampered by differing approaches to classify AMD. In our AugUR study survey, recruiting residents from in/around Regensburg, Germany, aged 70+, we analyzed the AMD status derived from color fundus images applying two different classification systems. Based on 1,040 participants with gradable fundus images for at least one eye, we show that including individuals with only one gradable eye (n = 155) underestimates AMD prevalence and we provide a correction procedure. Bias-corrected and standardized to the Bavarian population, late AMD prevalence is 7.3% (95% confidence interval = [5.4; 9.4]). We find substantially different prevalence estimates for “early/intermediate AMD” depending on the classification system: 45.3% (95%-CI = [41.8; 48.7]) applying the Clinical Classification (early/intermediate AMD) or 17.1% (95%-CI = [14.6; 19.7]) applying the Three Continent AMD Consortium Severity Scale (mild/moderate/severe early AMD). We thus provide a first effort to grade AMD in a complete study with different classification systems, a first approach for bias-correction from individuals with only one gradable eye, and the first AMD prevalence estimates from a German elderly population. Our results underscore substantial differences for early/intermediate AMD prevalence estimates between classification systems and an urgent need for harmonization.

## Introduction

Age-related macular degeneration (AMD) is a degenerative disorder affecting the choroid/retinal pigment epithelium (RPE)/photoreceptor complex of the central retina and represents the leading cause of central vision loss in the elderly populations of industrialised countries^1,2^. The irreversible, vision impairing clinical endpoint is late AMD, which can appear as a neovascular (NV) complication characterised by choroidal/sub-retinal ingrowth of diseased blood vessels or an atrophic form known as geographic atrophy (GA)^2^. Therapeutic options are limited^3,4^. Late AMD is typically preceded by clinically asymptomatic stages of early or intermediate AMD. These early/intermediate stages are determined by differently sized yellowish accumulations of extracellular material (drusen) between Bruch’s membrane and RPE or between RPE and the neurosensory retina. Other features of early/intermediate AMD are RPE abnormalities, including depigmentation or increased amount of pigment^2^.

Early/intermediate and late AMD stages can be documented by color fundus images of the central retina. While the definition of late AMD is reasonably homogeneous across classification systems, these systems differ substantially in their approach to classify early/intermediate AMD: utilized systems include the Wisconsin age-related maculopathy grading system (WARMGS)^5^, the Rotterdam Study classification^6^, or two AMD severity scales developed by the Age-Related Eye Disease Study (AREDS, 9-step Severity Scale and Simplified Severity Scale)^7,8^, all based on different combinations of presence, type, size and/or area of drusen as well as pigmentary changes. The two most recently established classification systems for early/intermediate AMD designed to capture risk of progression to late AMD are (i) the Clinical Classification from 2013^9^, assessing the presence of large drusen and/or pigmentary abnormalities^9^; and (ii) the Three Continent AMD Consortium Severity Scale from 2014^10^, additionally factoring in drusen size and area^10^.

The different classification systems used across studies hamper the comparison of published prevalence estimates for early/intermediate stages of AMD and there is little knowledge about the extent of the discrepancies. A first and notable effort in the year 2014 was made by the Three Continent AMD Consortium^10^ re-grading 60 images in each of four studies, which highlighted substantial differences in prevalence estimates before and after the application of a harmonized classification system (the above noted Three Continent AMD Consortium Severity Scale)^10^. Still, a comparison with the Clinical Classification or any other system in a full study has been lacking. A further issue that can induce misclassification and between-study differences is the inclusion of participants with only one gradable eye, which is common practice in ophthalmic epidemiology^5,7–11^: defining a person’s AMD status as the AMD status in the worse eye implies a larger probability of AMD “diagnosis” when there are gradable images for both eyes compared to only one eye. However, there is no evaluation of the extent of this bias or any approach to correct for it.

Moreover, data from Germany or Central Europe on late and early/intermediate AMD prevalence is limited, as emphasized by the German Ophthalmology Society^12^: the only two studies from Germany, the Gutenberg Health Study (GHS, n = 4,340)^13^ and the Cooperative Health Research in the Augsburg Region (KORA) study (n = 2,546)^11^, provide estimates in a general population aged 35–74 or 25–75 years, respectively, and include as few as nine or six late AMD patients, respectively^11,13^. A recent meta-analysis of 39 population-based studies worldwide includes only one population-based study from Central Europe (the Rotterdam Study)^14^.

We thus set out to classify AMD status in a full study with the two most recently developed classification systems, based on the cross-sectional survey of our AugUR study platform (Age-related diseases: understanding genetic and non-genetic influences - a study at the University of Regensburg), where we recruit participants at the age of 70+, who are able and willing to come to the study center. We here present prevalence estimates for early/intermediate and late AMD from 1040 participants of this mobile elderly population and evaluate the dependency on different classification systems and different approaches to handle participants with only one gradable eye.

## Subjects and Methods

### Study population and study sample

AugUR is a research platform recruiting from the general mobile elderly population, for which we here present results from the baseline survey conducted in 2013–2015. The study protocol and recruitment procedures have been described previously^15^. Briefly, inhabitants of the city and county of Regensburg, Germany, with ≥70 years of age, were identified by local registries and invited to the study centre at the Regensburg University Hospital. The study region captures about 330.000 inhabitants of mostly Caucasian ethnicity. Individuals were included into the AugUR study, if they were able and willing to come to the study centre, to be subjected to a three-hour study program, and to provide informed written consent. By this recruiting strategy, our study sample is drawn from a mobile elderly population from Bavaria including urban and rural areas.

Among the 5,644 contactable individuals (2,437 men, 3,207 women) to which invitation letters were sent out for the baseline survey, 2,457 individuals answered to our invitation letter or the written reminder (43.5% contact proportion, Supplementary Table 1). Among these, 1,133 were able and willing to participate (46.1% cooperation proportion) yielding an overall response of 20.1%, which was higher in men than in women (25.5% versus 15.9%) and decreasing by age (26.0% in age group 70–74, 7.2% in those aged 90–95 years).

The study protocol was approved by the Ethics Committee of the University of Regensburg, Germany (vote 12-101-0258). The study complies with the 1964 Helsinki declaration and its later amendments.

### Assessment of participant data and color fundus images

Via a standardized face-to-face interview and medical exams, we gathered information on smoking, metabolic parameters (body-mass-index [BMI], type 2 diabetes mellitus [T2DM], hypertension) as well as ocular comorbidities (cataract, glaucoma, diabetic retinopathy) by trained medical staff (details in Supplementary Text 1). The Askimed software (http://www.askimed.com/) and SAS were used for data management.

Color fundus photography of the central retina was conducted using the automatized DRS camera (Digital Retinography System; CenterVue, Padova, Italy)^15^. This camera type had also been selected for the German NaKo study after careful evaluation in a pilot assessment^16^. At least two color fundus images of each eye were acquired capturing the central or the central nasal field of the retina within a 45° view, including the full macular region and optic disc (details in Supplementary Text 2).

Images were defined as gradable, if they fulfilled the following quality criteria allowing for the assessment of AMD: sufficient brightness and color contrast as well as full macular region captured. Images were excluded from AMD grading, if they revealed obscuring lesions (e.g. cataract) or lesions considered to be the result of a competing retinal disease hampering AMD grading (such as advanced diabetic retinopathy, high myopia, trauma, congenital diseases, or photocoagulation unrelated to choroidal neovascularization).

Gradable images were examined by an experienced and trained ophthalmological consultant (C.B.); questionable findings were discussed with a second trained grader (ophthalmological consultant, T.B.). To assess inter-rater reliability, the second grader performed an independent grading for 40% of the images (n = 450).

### AMD classification

In order to assess AMD features for each eye, the presence of drusen, pigment abnormalities (hyperpigmentation or depigmentation), GA or NV was determined using the gradable color fundus images (details in Supplementary Text 3).

To classify AMD, we applied the two most recently established classification systems: (i) the Clinical Classification^9^, designed by the Beckman Initiative for Macular Research Classification Committee to be especially usable for clinical routine, which distinguishes between early and intermediate AMD depending on the presence of large drusen and/or pigmentary abnormalities^9^; (ii) the Three Continent AMD Consortium Severity Scale^10^, developed by harmonizing the grading of the population-based Rotterdam Study, the Beaver Dam Eye Study, the Los Angeles Latino Eye Study and the Blue Mountain Eye Study, which separates mild early from moderate or severe early AMD stages depending on drusen size, drusen area, or the presence of pigmentary abnormalities^10^. These two classification systems differ in how they define “early” or “intermediate” AMD, both define late AMD as presence of GA and/or NV and describe five AMD categories in total (details in Supplementary Tables 2 and 3).

The Clinical Classification is based on data from the ARED Study and their strategy of a 9-step severity scale, combining a 6-step drusen area scale with a 5-step pigmentary abnormality scale for early AMD grading^7^. This 9-step severity scale has been further refined to the Simplified Severity Scale^8^, focusing on the presence of large drusen and/or pigmentary abnormalities as the main features of early AMD^8,17^. The Simplified Severity Scale was developed to predict the risk for the second eye to develop late AMD, i.e. the risk for the patient to suffer from total, binocular vision loss, rather than to predict the risk of late AMD in any eye. In this respect, this classification system is not comparable to the procedures of the Clinical Classification or the Three Continent AMD Consortium Severity Scale and was therefore not included in our analysis. Earlier developed classification systems like the WARMGS^5^ were also not included, because they considered e.g. drusen morphology (soft/indistinct or hard/distinct drusen), a rather subjective classification criterion. Instead, newer AMD classification systems rely on the well demarcated, measurable and more objective drusen size or area.

The AMD status of a participant was derived as the AMD status of the eye with the more severe AMD stage (“worse eye”) when both eyes were gradable, and as the grade of the one available eye otherwise. Participants with gradable images for at least one eye were included in the analysis.

### Statistical analyses

All statistical analyses were carried out using the statistical software package IBM SPSS Statistics, Version 23 (IBM, New York, USA) and R, Version 3.3.2 (R Core Team, 2016). To test for differences between the characteristics of the analyzed participants and the total sample, we utilized linear or logistic regression.

For each of the two classification systems, we derived the relative frequencies of AMD status as a five-category scale (Clinical Classification: no AMD, age-related changes, early, intermediate, late AMD; Three Continent AMD Consortium Severity Scale: no AMD, mild, moderate, severe early, or late AMD) and a three-category scale (no AMD, “early” or “intermediate” AMD, late AMD). Differences of relative AMD frequencies between men and women or a trend by age were tested by logistic regression using likelihood ratio tests. We derived standardized AMD prevalence estimates as the sum of relative frequencies by sex and five-year age-groups weighted for the proportion of the Bavarian population in the respective sex- and age-group (www.statistik.bayern.de/statistik/zensus/00843.php). Inter-grader agreement and inter-system agreement (using the three-category scale) was assessed by quadratic weighted Kappa (Fleiss)^18^.

### Correcting prevalence estimates for the bias from participants with only one gradable eye

While it is common practice to utilize the worse eye for participants with both eyes gradable (“two-eye participants”)^5,7–11^ and the only available eye otherwise (“one-eye participants”), the resulting AMD status of a one-eye participant is misclassified towards a lower disease stage, when the ungraded eye is worse than the observed eye. Thus, one-eye participants give rise to a bias in prevalence estimates.

We quantified this bias, explored the missing mechanism, and provided a correction procedure to obtain unbiased prevalence estimates for each of the disease stages $k$. Here, $k\,\in [1,\ldots ,5]$ for the 5-category scales (no, age-related changes, early, intermediate, late AMD for the Clinical Classification, or no, mild early, moderate early, severe early, late AMD for the Three Continent AMD Consortium Severity Scale, respectively) or $k\in [1,\ldots ,3]$ for the 3-category scale (no, any early/intermediate, late AMD). The general idea is to combine the (unbiased) estimate of disease stage $k$ of the two-eye participants with the bias-adjusted estimate from the one-eye participants weighted by the proportion of two- and one-eye participants in the study. The bias-adjustment is derived from the two-eye participants by computing the probability that a person with an “observed” disease stage l (using one randomly selected eye) has in fact a “true” disease stage $k$ (given by the worse eye), with $k,l\,\in [1,\ldots ,5]$ or $k,l\,\in [1,\ldots ,3]$. These probabilities (predictive values, ${\lambda }_{kl}$) are then applied as adjustment factors to the estimates from the one-eye participants. This works under the assumption that the missing eye of a one-eye participant is missing randomly with respect to the participant’s (true) AMD disease stage.

A detailed description of the approach can be found in Supplementary Text 4. When the study sample is not a random sample of the study population, the bias-adjustment can be applied to the relative disease stage frequencies by age-group and sex and the bias-adjusted disease stage frequencies by age-group and sex can then be used for standardization to yield bias-adjusted prevalence estimates (see also Supplementary Text 4). In the following, we present bias-corrected relative disease stage frequencies and bias-corrected AMD prevalence estimates that are standardized to the Bavarian population. We do this for each of the two AMD classification systems.

## Results

### Participant characteristics

Of the 1,133 AugUR participants, color fundus images were successfully acquired for at least one eye for 1,129 individuals. Of those, 1,040 participants had gradable images for at least one eye (92.1% compared to those with acquired images). The 1,040 participants included more men than women (54.2% versus 45.8%) and a mean age of 77.5 years (standard deviation 5.1 years, age ranging from 70 to 95 years). Other subject characteristics were in line with the high age of our participants (Table 1).

**Table 1:** Participant characteristics.

	All	Men	Women	N with non-missing values
Age [years], mean ± SD	77.5 ± 5.1	77.8 ± 5.1	77.2 ± 5.0	1040
**Lifestyle factors**				
Current smoker^a^, % (n)	6.2 (64)	7.3 (41)	4.4 (23)	1040
Ex-smoker^a^, % (n)	37.8 (393)	52.3 (295)	20.6 (98)	1040
Pack years^b^, mean ± SD	27.8 ± 32.0	29.9 ± 32.9	22.0 ± 24.0	1036
**Metabolic parameters**				
BMI^c^ [kg m^−²^], mean ± SD	28.0 ± 4.5	28.1 ± 4.0	27.8 ± 5.0	1037
T2DM^d^, % (n)	20.8 (216)	21.3 (120)	20.2 (96)	1040
Hypertension^e^, % (n)	72.8 (744)	72.5 (408)	73.2 (347)	1037
**Eye-related parameters**				
Cataract^f^, % (n)	48.7 (506)	41.0 (231)	57.8 (275)	1040
Cataract surgery^g^, % (n)	69.4 (351)	66.7 (154)	71.6 (197)	506
Glaucoma^f^, % (n)	7.1 (74)	7.3 (41)	6.9 (33)	1040
Diabetic retinopathy^f^, % (n)	1.1 (11)	1.4 (8)	0.6 (3)	1040
Pupil size^h^ [mm], mean ± SD	3.7 ± 0.7	3.6 ± 0.7	3.7 ± 0.7	1040

Shown are characteristics for the 1,040 analyzed participants from our AugUR study survey (564 men, 476 women).

Abbreviations: SD = standard deviation; BMI = body-mass-index; T2DM = type 2 diabetes;

^a^Currently smoking ≥1 cigarette per month; having stopped smoking for ≥1 month.

^b^Pack years are defined as number of packs (20 cigarettes per pack) smoked per day times the number of years of smoking, estimating that the participant has started smoking at the age of 18 years.

^c^BMI is defined as measured weight divided by squared measured body height.

^d^T2DM is defined as a self-reported diagnosis or anti-diabetes medication intake.

^e^Hypertension is defined as actually measured systolic blood pressure of ≥140 mmHg, diastolic blood pressure of ≥90 mmHg or corresponding medication taken, given that the participants were aware of having hypertension.

^f^History of cataract, glaucoma and diabetic retinopathy was assessed via self-report.

^g^History of cataract surgery was assessed via self-report among those with reported cataract (n = 506).

^h^Pupil size per person is defined by the smaller pupil diameter of both eyes.

The 93 participants without any gradable eye (n = 4 without any acquired image, n = 89 with acquired but non-gradable image for any eye) were older than the 1,040 analyzed subjects (mean age = 79.0 versus 77.5 years, adjusted P = 0.01, Supplementary Table 4). The 89 non-gradable individuals also differed from the 1,040 analyzed subjects by a smaller average pupil size (mean pupil size 2.7 versus 3.7 mm, adjusted P = 3.3 * 10^−23^) and by a smaller proportion with pharmacological mydriasis (48.3% versus 63.0%, adjusted P = 0.01). None of the other participant characteristics differed when adjusting for age and sex (adjusted P ≥ 0.05, Supplementary Table 4).

### Prevalence of late AMD

Among the 1,040 analyzed participants, late AMD was detected in 63 individuals (6.1%, Table 2; Supplementary Table 5), with no difference between the two classification systems by definition. Among these 63 participants, 16 revealed pure GA in the same or the other eye (25.4%) including 7 with pure GA in both eyes, 31 showed pure scaring/NV in the same or the other eye (49.2%) including 8 with scaring/NV in both eyes, and 16 participants had mixed GA and scaring/NV in one or both eyes (25.4%).

**Table 2:** Observed frequencies of AMD status and prevalence estimates for two classification systems.

	Observed frequencies	Prevalence^a^ [95% CI]
**Clinical Classification**		
No AMD, no apparent aging changes, %	27.5	25.8 [23.0; 28.5]
No AMD, normal aging changes, %	23.0	22.8 [20.0; 25.6]
Early AMD, %	26.4	27.5 [24.5; 30.6]
Intermediate AMD, %	17.0	16.7 [14.2; 19.2]
Late AMD, %	6.1	7.2 [5.3; 9.1]
**Three Continent AMD Consortium Severity Scale**		
No AMD, %	77.1	76.3 [73.4; 79.2]
Mild early AMD, %	8.9	8.8 [7.0; 10.7]
Moderate early AMD, %	4.2	4.2 [2.9; 5.5]
Severe early AMD, %	3.7	3.5 [2.3; 4.8]
Late AMD, %	6.1	7.2 [5.3; 9.1]

Shown are the observed frequencies and the standardized prevalence estimates (using the Bavarian population) for each AMD status based on the Clinical Classification^9^ and the Three Continent AMD Consortium Severity Scale^10^ in the 1,040 analyzed individuals (at least one eye gradable). Details by sex and age-group are shown in Supplementary Tables 5 and 6.

Abbreviations: AMD = age-related macular degeneration; CI = confidence interval;

^a^Five-year-age group- and sex-standardized prevalence estimates based on the weights from the Bavarian population and corresponding 95% confidence intervals.

Using the Bavarian population over 70 years of age as a reference, age- and sex-standardized prevalence for late AMD was estimated as 7.2% (95% confidence interval = [5.3; 9.1], Table 2; Supplementary Table 6). There was a substantial increase of late AMD prevalence by age-group (sex-standardized prevalence from ~2% to 19% for those aged 70–74 to those at 90+; logistic regression controlled for sex, likelihood-ratio test P < 0.001), but no significant difference by sex (logistic regression controlled for age-group, P = 0.35). The apparent difference between prevalence estimates of 6.0% (95%-CI = [3.0, 8.9]) for men and 8.0% (95%-CI = [5.1, 11.0]) for women was due to the higher age of the women.

### Prevalence of “early” and “intermediate” AMD

The frequencies of early or intermediate AMD stages vary between the two classification systems: When applying the Clinical Classification, we observed 277 out of the 1,040 participants (43.5%) with early or intermediate AMD; when considering the Three Continent AMD Consortium Severity Scale, 175 participants (16.8%) had any form of early AMD (mild, moderate, or severe; Table 2; Supplementary Table 5).

Based on the Bavarian population, we derived age- and sex-standardized prevalence estimates: For the Clinical Classification, prevalence was estimated as 27.5% (95%-CI = [24.5; 30.6]) for early and 16.7% (95%-CI = [14.2; 19.2]) for intermediate AMD, totalling 44.2% for any early/intermediate AMD. For the Three Continent AMD Consortium Severity Scale, prevalence was 8.8% (95%-CI = [7.0; 10,7]) for mild, 4.2% (95%-CI = [2.9; 5.5]) for moderate, and 3.5% (95%-CI = [2.3; 4.8]) for severe early AMD, thus a total of 16.5% for any early AMD (Table 2; Supplementary Table 6). This highlights a substantial difference in the prevalence estimates of “early” or”intermediate” AMD stages when comparing these two systems.

There was no significant increase in the prevalence by five-year age-group for any type of “early” or “intermediate” AMD using logistic regression controlled for sex. Controlled for age, there was no difference in the prevalence by sex for any type of early or intermediate AMD, except for early AMD based on the Clinical Classification with a higher odds of early AMD in women (P = 0.003, Supplementary Table 6). AMD features per eye (i.e. drusen, pigmentary abnormalities, details on GA or NV) are described in Supplementary Table 7 and Supplementary Text 5.

### Discordance between classification systems and concordance between graders

To understand the differences between the two classification systems, we cross-tabulated participants’ AMD status as derived by each of the systems and found an interesting pattern to emerge (Table 3): (i) the Clinical Classification “intermediate” AMD is almost completely consistent with “any early” AMD of the Three Continent AMD Consortium Severity Scale (mild/moderate/severe): 175 of 177 participants with “intermediate” AMD have “any early” AMD; all of the “any early” AMD individuals are classified as “intermediate”, (ii) the “no AMD” category of the Three Continent AMD Consortium Severity Scale is differentiated by the Clinical Classification as “no AMD”, “age-related changes”, and “early AMD”. Thus, we see a more refined differentiation of the Three Continent AMD Consortium Severity Scale “no AMD” when applying the Clinical Classification and a refinement of the Clinical Classification “intermediate AMD” when applying the Three Continent AMD Consortium Severity Scale. (iii) Importantly, the Clinical Classification yields 277 more individuals (out of the 1040 total) as “early/intermediate” AMD compared to the Three Continent AMD Consortium Severity Scale. All these 277 persons were graded as “no AMD” when applying the Three Continent AMD Consortium Severity Scale. This substantiates the fact that the Clinical Classification is more inclusive in its approach to grade early/intermediate AMD compared to the Three Continent AMD Consortium Severity Scale.

**Table 3:** Discordance across the applied classification systems in a five-category interpretation.

		Three Continent AMD Consortium Severity Scale					Total, n
		No AMD, n	Mild early AMD, n	Moderate early AMD, n	Severe early AMD, n	Late AMD, n	
Clinical Classification	No AMD, no apparent aging changes, n	286	0	0	0	0	286
	No AMD, normal aging changes, n	239	0	0	0	0	239
	Early AMD, n	275	0	0	0	0	275
	Intermediate AMD, n	2	93	44	38	0	177
	Late AMD, n	0	0	0	0	63	63
Total, n		802	93	44	38	63	1040

Shown are cross-tabulated number of participants by AMD status for the Clinical Classification^9^ and the Three Continent AMD Consortium Severity Scale^10^ using the five-category scale.

Abbreviations: AMD = age-related macular degeneration.

Since the five AMD categories cannot be matched 1:1 between the two systems, we cannot compare agreement there, but rather in a three-category interpretation: When collapsing the early/intermediate AMD and the mild/moderate/severe early AMD to an “any early or intermediate AMD” category yielding a 3 × 3 concordance table (Table 4), we found a substantial proportion of participants discordant between the two classification systems (27.6%) and only a modest agreement (quadratic weighted kappa = 0.652).

**Table 4:** Discordance across the applied classification systems in a three-category interpretation.

		Three Continent AMD Consortium Severity Scale			
		No AMD, n	“Any early” AMD^b^, n	Late AMD, n	Total, n
**Clinical Classification**	No AMD, n	525	0	0	525
	“Any early or intermediate” AMD^a^, n	277	175	0	452
	Late AMD, n	0	0	63	63
**Total, n**		802	175	63	1040

Shown are cross-tabulated number of participants by AMD status for the Clinical Classification^9^ and the Three Continent AMD Consortium Severity Scale^10^ using the three categories “no AMD”, “any early or intermediate AMD”, and “late AMD”.

Abbreviations: AMD = age-related macular degeneration;

^a^For the Clinical Classification collapsing early AMD and intermediate AMD.

^b^For the Three Continent AMD Consortium Severity Scale, collapsing mild early AMD, moderate early AMD, and severe early AMD to “any early” AMD.

While it is not clear which of the two classification systems would be regarded as a gold standard, a clear criterion for judging the quality of a system is a low inter-rater variability. We graded images for 450 participants by two graders, for each of the two classification systems, and found good concordance/agreement for both systems (concordance = 83.9% or 95.3%, quadratic weighted kappa = 0.902 or 0.972, for the Clinical Classification or the Three Continent AMD Consortium Severity Scale, respectively, Supplementary Tables 8 and 9). In summary, the two classification systems provide reliable results within each system, but not across the systems.

### Underestimation of AMD status due to missing eyes

To derive the participants’ AMD status in epidemiological studies, the worse eye is used for participants with gradable images for both eyes (n = 855, 82.2% of analyzed subjects, “two-eye participants”) and the available eye for participants with gradable images for only one eye (n = 155, 14.9%, “one-eye participants”). For both classification systems, we observed lower relative AMD frequencies among the 155 one-eye participants compared to the 885 two-eye participants (e.g. late AMD: 4.5% versus 6.3%; Clinical Classification “early/intermediate AMD”: 34.2% versus 45.1%; Three Continent AMD Consortium Severity Scale “any early AMD”: 10.3% versus 18.0%, Tables 5 and 6). Also the relative AMD frequencies among all participants (at least one eye gradable, n = 1,040), as a sample-size weighted average of two-eye and one-eye participants’ frequencies, are lower than they would be when both eyes would have been gradable for all.

**Table 5:** Bias-corrected relative frequencies and prevalence estimates by AMD status for two classification systems in a five-category interpretation.

	Naïve frequencies			Bias-corrected frequencies		Bias-corrected prevalence^a^
	Both eyes gradable n = 885	Only one eye gradable n = 155	All n = 1040	Only one eye gradable n = 155	All n = 1040	All n = 1040
**Clinical Classification**						
No AMD, no apparent aging changes, %	25.6	38.1	27.5	27.3	25.9	24.3 [21.5; 27.1]
No AMD, normal aging changes, %	22.9	23.2	23.0	24.9	23.2	23.1 [20.2; 26.1]
Early AMD, %	26.9	23.9	26.4	27.6	27.0	27.9 [24.8; 31.1]
Intermediate AMD, %	18.2	10.3	17.0	14.4	17.6	17.4 [14.9; 19.9]
Late AMD, %	6.3	4.5	6.1	5.7	6.2	7.4 [5.4; 9.4]
**Three Continent AMD Consortium Severity Scale**						
No AMD, %	75.7	85.2	77.1	79.9	76.3	75.5 [72.5; 78.5]
Mild early AMD, %	9.3	7.1	8.9	9.7	9.3	9.2 [7.3; 11.2]
Moderate early AMD, %	4.6	1.9	4.2	3.0	4.4	4.4 [3.1; 5.8]
Severe early AMD, %	4.1	1.3	3.7	1.8	3.7	3.6 [2.4; 5.0]
Late AMD, %	6.3	4.5	6.1	5.5	6.2	7.3 [5.4; 9.4]

Shown are naïve relative frequencies of observed AMD stages among the participants with both eyes gradable (n = 885, worse eye), among the participants with one eye gradable (n = 155), and all analyzed participants (at least one eye gradable, n = 1040, worse eye or only available eye). We also show the bias-corrected frequencies and bias-corrected prevalence estimates using predictive values (Supplementary Text 4; Supplementary Table 12). Results are shown for the Clinical Classification^9^ and the Three Continent AMD Consortium Severity Scale^10^ for the five-category scale. Details by sex and age-group are shown in Supplementary Tables 14 and 15.

Abbreviations: OD = right eye; OS = left eye; AMD = age-related macular degeneration;

^a^Prevalence estimates standardized by five-year-age group and sex based on the weights from the Bavarian population.

**Table 6:** Bias-corrected relative frequencies and prevalence estimates by AMD status for two classification systems in a three-category interpretation.

	Naïve frequencies			Bias-corrected frequencies		Bias-corrected prevalencea
	Both eyes gradable n = 885	Only one eye gradable n = 155	All n = 1040	Only one eye gradable n = 155	All n = 1040	All n = 1040
**Clinical Classification**						
No AMD, %	48.6	61.3	50.5	52.3	49.1	47.4 [44.0; 50.7]
“Any early or intermediate” AMD^b^, %	45.1	34.2	43.5	41.8	44.6	45.3 [41.8; 48.7]
Late AMD, %	6.3	4.5	6.1	5.9	6.3	7.4 [5.4; 9.4]
**Three Continent AMD Consortium Severity Scale**						
No AMD, %	75.7	85.2	77.1	79.9	76.3	75.5 [72.5; 78.5]
“Any early” AMD^c^, %	18.0	10.3	16.8	14.3	17.4	17.1 [14.6; 19.7]
Late AMD, %	6.3	4.5	6.1	5.7	6.2	7.3 [5.4; 9.4]

Shown are naïve relative frequencies of observed AMD stages among the participants with both eyes gradable (n = 885, worse eye), among the participants with one eye gradable (n = 155), and all analyzed participants (at least one eye gradable, n = 1040, worse eye or only available eye). We also show the bias-corrected frequencies and bias-corrected prevalence estimates using predictive values (Supplementary Text 4; Supplementary Table 13). Results are shown for the Clinical Classification^9^ and the Three Continent AMD Consortium Severity Scale^10^ for the three categories “no AMD”, “any early or intermediate AMD”, and “late AMD”. Details by sex and age-group are shown in Supplementary Tables 14 and 15.

Abbreviations: OD = right eye; OS = left eye; AMD = age-related macular degeneration.

^a^Prevalence estimates standardized by five-year-age group and sex based on the weights from the Bavarian population.

^b^For the Clinical Classification collapsing early AMD and intermediate AMD.

^c^For the Three Continent AMD Consortium Severity Scale, collapsing mild early AMD, moderate early AMD, and severe early AMD to “any early” AMD.

To evaluate the missing mechanism, we analyzed the disease stage frequencies based on single eyes of the two-eye participants (Supplementary Tables 10 and 11) and found the following: (i) there were no differences in relative AMD frequencies when using right compared to left eyes (Stuart-Maxwell Test P = 0.33 or 0.29 for the Clinical Classification or the Three Continent AMD Consortium Severity Scale, respectively); (ii) AMD frequencies were lower when only one eye was utilized instead of the worse eye; (iii) a Monte-Carlo style evaluation (re-sampling 155 participants out of the two-eye participants and computing the distribution of AMD frequencies of randomly selected single eyes) yielded relative AMD frequencies similar to those observed for one-eye participants (data not shown). These observations are in line with the assumption of randomly missing eyes.

In order to derive bias-corrected AMD frequencies and variance estimates, we computed the predictive values (see Methods; Supplementary Tables 12 and 13). We then applied these predictive values to derive bias-corrected AMD frequencies by sex and five-year age-group, weighted these estimates according to the Bavarian population, and derived bias-corrected standardized prevalence estimates: for the Clinical Classification, corrected “early/intermediate AMD” prevalence was 45.3% (95%-CI = [41.8; 48.7]) instead of the naïve 44.2%; for the Three Continent Consortium, corrected prevalence of “any early” AMD was 17.1% (95%-CI = [14.6; 19.7]) instead of the naïve 16.5%; for late AMD, corrected prevalence was 7.4% (Clinical Classification) and 7.3% (Three Continent Consortium) (95%-CI = [5.4; 9.4]) instead of 7.2%, (Tables 5 and 6; Supplementary Tables 14 and 15**)**. Thus, including one-eye participants underestimates prevalence and we provide a correction procedure for this bias.

## Discussion

Our data and results emphasize substantial differences in prevalence estimates of early/intermediate AMD between the two classification systems that we have applied to all of our 1,040 study participants: The Clinical Classification yields a prevalence for “early/intermediate AMD” that is more than twice as high compared to the Three Continent AMD Consortium Severity Scale. Moreover, we provide a first approach to correct prevalence estimates for the bias, which results from including individuals with only one gradable eye. Finally, while we acknowledge our focus on a “mobile elderly” rather than a full elderly population, we here provide the first prevalence estimates of early/intermediate and late AMD in a German elderly population.

Our finding of twice as high prevalence estimates using one versus the other classification system has both epidemiological and clinical implications. Epidemiologically, this underscores that the comparison between and the joint analysis of studies applying different classification systems is severely hampered. For clinical practice, it still remains unclear how to classify early/intermediate AMD when one system yields twice as many diagnosed individuals compared to the other. Our data and results fuel an ongoing debate on how to classify AMD stages^10^. Earlier attempts to harmonization resulted in the Three Continent AMD consortium Severity Scale^10^ highlighting substantially different prevalence estimates using the harmonized classification compared to the studies’ originally applied classification, which were the WARMGS^5^ and the Rotterdam Study classification^6^. In parallel, the Clinical Classification system^9^ was established, but any comparison between these two most recently developed systems has been lacking. The substantial differences that we find highlight a need to continue working towards a uniform AMD classification.

Our cross-tabulation shows that the Clinical Classification refines the “no AMD” group from the Three Continent Consortium Severity Scale into “no changes”, “age-related changes” and “early AMD”, while the latter refines the Clinical Classification’s “intermediate AMD” into “mild/moderate/severe early AMD”. This supports the notion that the Clinical Classification tries to not miss any symptoms, while the Three Continent Consortium Severity Scale focusses on the more pronounced stages. The question arises what is relevant for progression to late AMD: is the Clinical Classification more thorough or does the Three Continent Consortium Severity Scale have a better focus? To answer this, longitudinal studies are warranted. To ascertain power and to cover potentially heterogeneous effects from different life style factors, these studies will need to include a substantial number of elderly individuals, implement a long follow-up period to allow for late AMD to evolve, and cover various geographical regions with different life styles.

Moreover, we were interested in evaluating the common epidemiological practice to include participants with images available/gradable for both eyes (“two-eye participants”) as well as those with only one gradable eye (“one-eye participants”), using the worse eye to classify the person’s AMD stage^10,19,20^. Our results clearly show that the inclusion of one-eye-participants introduces a bias in the sense that it underestimates disease prevalence. This is due to the fact that there is an additional chance to detect AMD when we can look at the second eye rather than when there is only one eye gradable. We here provide a procedure to correct for this bias. The alternative, excluding one-eye participants, would diminish power unnecessarily. We feel that this bias might have been under-acknowledged so far. Despite the fact that the extent of the bias is rather small when one-eye participants constitute only a relatively small fraction of the study sample (~15% here), our procedure provides an easy approach to correct for this bias so there is no reason to ignore.

We would like to emphasize that the bigger the fraction of one-eye participants, the bigger the bias from ignoring the missing disease grading. Therefore, our bias correction will become more important with larger proportions of one-eye participants in a study sample. To illustrate this, we conducted some example calculations: Let’s assume that the “true” AMD prevalence (given by the AMD status in the worse eye) in a population is 75.5%, 17.1%, and 7.3% for no, any early, or late AMD (as estimated here in the AugUR study based on the Three Continent AMD Consortium Severity Scale, Table 6). If the study is a random sample from this population and if the study has zero one-eye participants, we can expect to observe the “true” prevalence estimates of 75.5%, 17.1%, and 7.3%. Based on the derived predictive values (Supplementary Table 13), we can quantify the bias for different fractions of one-eye participants: For 15% (as in AugUR), we would expect naïve (not bias-adjusted) AMD prevalence estimates of 76.2%, 16.6%, and 7.1%, which equals a multiplicative bias of 1.01, 0.97, and 0.97 compared to the true prevalence. For 30% (50%), we would expect naïve estimates of 77.0%, 16.0%, and 6.9% (78.0%, 15.3%, 6.6%) and the multiplicative bias is 1.02, 0.94, and 0.94 (1.03, 0.90, 0.90). For a study with 100% one-eye participants, we would expect naïve prevalence estimates of 80.5%, 13.6%, and 5.8% yielding a multiplicative bias of 1.06, 0.79, and 0.79. These results are based on the assumption that the predictive values are constant in the population and the missing eyes in the one-eye participants are missing independently of the disease stage in the eyes. For a population with different disease prevalence and different predictive values, the magnitude of bias from ignoring misclassification in one-eye participants can deviate from the above given numbers. For such situations, a sample of two-eye participants in necessary, which has to be sufficiently large to obtain stable estimates of the predictive values. The proposed bias-correction will also increase the comparability between studies with different fractions of one-eye participants. Thus, we recommend this bias-correction for further epidemiological studies.

We compared AugUR late AMD prevalence estimates with other cross-sectional, previously published studies of Caucasian/European ancestry (Supplementary Table 16). Contrasting our data with estimates from other studies in Germany is limited due to scarce data in individuals at the age of 70+, which is the age when late AMD is most prevalent. The GHS^13^ and the KORA study^11^ are based on general adults including only a limited number of participants at higher age: the ~400 participants in GHS and 156 in KORA with 70–75 years of age, none with 75+, include nine or six individuals with late AMD, respectively, compared to our 1,040 AugUR participants including 63 individuals with late AMD. Internationally, there are a few studies with notable sample sizes also in the elderly that allow for early/intermediate and late AMD prevalence. For participants aged 75–84 years, which appears to be a good age-group to compare given published data across studies, our late AMD prevalence of 7.0% compares to 7.3% from Iceland (Age, Gene/Environment Susceptibility Reykjavik Study)^21^, 3.0% from the multicentre European Eye Study^1^, 3.7% from the Rotterdam Study^22^ 6.1% from a very recent study in England (Bridlington Eye Assessment Project)^23^, 2.2% from Ireland (The Irish Longitudinal study on Ageing)^24^, 5.4% from Australia (Blue Mountains Eye Study)^20^, and 7.1% from the USA (Beaver Dam Eye Study)^19^. For early/intermediate AMD, we would like to refrain from a comparison due to the above noted substantial differences in classification systems, but published numbers broken down by age-groups are provided in Supplementary Table 16.

Our study is population-based, which is a typical study design to capture prevalence data. We need to acknowledge the limitation that our response of 20.1% is low and does not allow for a generalization to the general elderly. Recruitment strategies for population-based studies, particularly for participants at high age, need to weigh in an extended study program in a study center (restricting to mobile participants) with a limited study program administered in home visits (less selection due to lacking mobility). Our recruitment strategy requires participants to be mentally and physically able and willing to come to the study center. We thus describe our study population as the “mobile elderly” who conduct a rather autonomous life-style and respond to health themes. It is difficult to judge to what extent the non-response in this AugUR survey is AMD-related: there might be participants that are hindered to respond due to vision impairment (underestimating late AMD prevalence), those that feel motivated to respond because of increased health awareness that usually comes with a healthy life-style (underestimating AMD prevalence when we assume a life-style component of the disease), or those who respond due to vision impairment in their families (overestimation). Since early AMD usually lacks any symptoms like vision impairment, the corresponding prevalence estimates should be less affected. A complementing survey from nursing homes with a restricted study program on-site might be a future add-on. Given that there are currently – to our knowledge – no prevalence estimates for early and late AMD in the elderly in Germany and given that each recruitment strategy has its pros and cons, the here presented prevalence estimates are an important contribution to understand the dimension of this disease.

A potential concern would be persons and eyes where the fundus image is ungradable due to features related to AMD. Since we have a high proportion of gradable participants among those where fundus imaging was conducted (92.1%), which we can attribute to our application of mydriasis for most participants, we would not assume a large potential of bias by AMD-related drop-outs due to image quality.

At this point, we further acknowledge that this present analysis and data is based on color fundus images only, and not on multimodal imaging, such as optical coherence tomography. One might perceive this as a limitation, as multimodal imaging is standard in clinical diagnostics of AMD and allows for a more detailed retinal assessment. However, there is currently no published epidemiological AMD classification system, especially for early/intermediate AMD, that integrates multimodal imaging.

Clear strengths of our study are the clear recruitment strategy, standard operating procedures for all aspects of the study, face-to-face interviews by trained study nurses, and stringent quality control. Strengths of our analysis include the detailed analyses of discrepancies between graders, between AMD classification systems, and between different approaches to handle participants with only one eye gradable.

In summary, we here provide prevalence estimates for AMD from a mobile elderly population of South-East Germany, filling a crucial gap of data on AMD in Germany and Central Europe. Our data underscores substantial differences in early/intermediate AMD prevalence with twice as high estimates using the Clinical Classification compared to the Three Continent AMD Consortium Severity Scale. Future efforts are required to work towards a unified system to enable between-study comparison and large-scale meta-analyses. The currently limited knowledge on the etiology of early/intermediate AMD and the progression factors to late AMD can be expected to flourish once these obstacles are resolved. This may, in the long term, help to further identify preventive or therapeutic options.

### Data availability statement

Access to individual data for specific research purposes can be obtained by contacting the corresponding author.

## Electronic supplementary material

Supplementary Information
