# Supplementary material for: On the impact of different approaches to classify age-related macular degeneration: Results from the German AugUR study
Source: Sci Rep. 2018 Jun 6;8:8675. doi: 10.1038/s41598-018-26629-5 (PMC5989235; doi:10.1038/s41598-018-26629-5)
Supplement: Supplementary file 1 — Supplementary Information [file 41598_2018_26629_MOESM1_ESM.pdf]

On the impact of different approaches to classify age-related macular degeneration: Results from the German AugUR study

Caroline Brandl[1,2,3], Martina E. Zimmermann[1], Felix Günther[4], Teresa Barth[2], Matthias Olden[1], Sabine C. Schelter[1,5], Florian Kronenberg[6], Julika Loss[7], Helmut Küchenhoff[4], Horst Helbig[2], Bernhard H. F. Weber[3], Klaus J. Stark[1], Iris M. Heid[1]*

1) Department of Genetic Epidemiology, University of Regensburg, Regensburg, Germany

2) Department of Ophthalmology, University Hospital Regensburg, Regensburg, Germany

3) Institute of Human Genetics, University of Regensburg, Regensburg, Germany

4) Statistical Consulting Unit StaBLab, Department of Statistics, Ludwig-Maximilians-University Munich, Germany

5) Centre for Clinical Studies, University Hospital Regensburg, Regensburg, Germany

6) Division of Genetic Epidemiology, Medical University of Innsbruck, Innsbruck, Austria

7) Medical Sociology, Institute of Epidemiology and Preventive Medicine, University of Regensburg, Regensburg, Germany

*) Corresponding Author:

Prof. Dr. Iris M. Heid

Department of Genetic Epidemiology, University of Regensburg

Franz-Josef-Strauss-Alle 11

93053 Regensburg, Germany

Tel.: +49 (0)941/944-5210

Fax: +49 (0)941/944-5212

Email: iris.heid@klinik.uni-regensburg.de

SUPPLEMENTARY INFORMATION

Contents

Supplementary Table 1.	Contact and response proportion in the AugUR baseline enrolment.
Supplementary Text 1.	Assessment of participant data.
Supplementary Text 2.	Acquisition and processing of color fundus images.
Supplementary Text 3.	AMD classification.
Supplementary Table 2.	Applied classification systems for AMD – the Clinical Classification.
Supplementary Table 3.	Applied classification systems for AMD – the Three Continent AMD Consortium Severity Scale.
Supplementary Text 4.	Correcting AMD prevalence estimates for the bias from participants with only one gradable eye.
Supplementary Table 4.	Characteristics of recruited and analyzed subjects from the AugUR study.
Supplementary Table 5.	Observed relative frequencies of AMD status for two classification systems by sex and five-year age-groups.
Supplementary Table 6.	Prevalence estimates of AMD for two classification systems by five-year age-groups.
Supplementary Table 7.	Frequencies of specific AMD features observed per eye in the AugUR study.
Supplementary Text 5.	AMD features per eye.
Supplementary Table 8.	Concordance of AMD status between two graders applying the Clinical Classification.

Supplementary Table 9. Concordance of AMD status between two graders applying the Three Continent AMD Consortium Severity Scale.

Supplementary Table 10. Evaluation of the effect of single eye grading based on the 885 participants with both eyes gradable in a five-category interpretation.

Supplementary Table 11. Evaluation of the effect of single eye grading based on the 885 participants with both eyes gradable in a three-category interpretation.

Supplementary Table 12. Predictive Values for the five-category scale.

Supplementary Table 13. Predictive Values for the three-category scale.

Supplementary Table 14. Bias-corrected observed relative frequencies for two classification systems by sex and five-year age-groups.

Supplementary Table 15. Bias-corrected prevalence estimates for two classification systems by five-year age-groups.

Supplementary Table 16. AMD prevalence estimates in participants 70+ in AugUR and previously published population-based studies.

References

Supplementary Table 1. Contact and response proportion in the AugUR baseline enrolment. Shown are the numbers of contactable persons and final number of study participants (n = 1,133) as well as the percentages relative to both, the drawn sample and the contactable sample. 327 individuals were non-contactable because they had died or moved away.

	Number	Drawn sample	Contactable sample
**Drawn random sample**	**5971**	**100%**	-
Died or moved away	327	5.5%	-
**Contactable persons**	**5644**	**94.5%**	**100%**
No contact[a]	3187	53.4%	56.5%
No participation (active refusal)	1324	22.2%	23.5%
**AugUR study participants**	**1133**	**19.0%**	**20.1%**

[a]) Did not answer the invitation letter nor a written reminder.

Supplementary Text 1. Assessment of participant data.

Smokers were categorized as current smokers (having smoked ≥ 1 cigarette per month), ex-smokers (having stopped smoking ≥ 1 month), and never smokers (having smoked less than 100 cigarettes in their lifetime). Pack years were calculated by multiplying the number of packs of cigarettes smoked per day with the number of years the person has smoked. The number of years of smoking was computed from the age at examination for current smokers or the age when smoking stopped for ex-smokers minus 18 under the assumption that smoking started around the age of 18.

The metabolic parameters, body-mass-index (BMI), type 2 diabetes mellitus (T2DM), and hypertension, were assessed by physical examination and interview information: BMI (kg/m2) was computed based on measured weight in kg (in light clothing, to nearest 0.1 kg) and height in m (to nearest 0.005 m) as weight divided by squared body height.[1,2] T2DM was assessed as self-reported type 2 diabetes or reported anti-diabetes therapy intake. Hypertension was assessed according to previous work [3,4] [5] as measured systolic blood pressure of ≥ 140 mmHg, diastolic blood pressure of ≥ 90 mmHg, or anti-hypertensive medication taken, given that the participants were aware of having hypertension.

A history of non-AMD related eye diseases such as cataract, glaucoma, or diabetic retinopathy was assessed via self-report during a standardised face-to-face interview.

Supplementary Text 2. Acquisition and processing of color fundus images.

Color fundus images of the central retina were acquired using the automatized DRS camera (Digital Retinography System; CenterVue, Padova, Italy). In line with the standard operating procedure of the NaKo study [6], we initially relinquished mydriasis for practical reasons. Consistent with previous observations that the quality of fundus photography depends on pupil size and that pupil size depends on age [7], we found the pupil size and thus the quality of fundus images to be insufficient for a substantial proportion of our elderly study cohort. We thus altered the protocol in January 2015 to administer a mild mydriasis (Mydriaticum UD, pharmaSTULLN, Stulln, Germany), after obtaining special written informed consent and explicit information about the consequences of mydriasis, such as a ban on driving and a small risk for acute angle-closure glaucoma (1 in 20,000 to 1 in 3,000)[8,9]. This risk was minimized by excluding participants from mydriasis that exhibited a flat anterior chamber, as assessed via a portable slit lamp examination (Kowa, Düsseldorf, Germany). For participants that were not eligible for mydriasis, fundus photography in miosis was attempted nevertheless, after waiting 5 minutes in darkened surroundings to allow pupils to dilate. Of note, pupil size was determined automatically by the DRS camera.

Color fundus images were exported as .jpg-files with a resolution of 2592 x 1944 pixels from the DRS camera. They were imported into the K-DRS software, a self-developed application for image analysis: images are displayed with a standardized front end on a 27 inch color-calibrated monitor, information on quality and grading can be entered, and results are linked with image number, participant identifier (IDGenerator [10]), and name of grader.

Supplementary Text 3. AMD classification.

For each eye, the presence of drusen and pigment abnormalities (hyperpigmentation or depigmentation) on color fundus images was assessed. Only lesions within 2 standard disc diameters (approx. 3000 μm) of the centre of the macula/fovea were considered. To determine drusen size category (small, intermediate, large), the smallest drusen diameter was compared to the width of a major branch retinal vein crossing the optic disc margin [11], considered to be approximately one-twelfth disc diameter (i.e. 125 μm, assuming the average disc diameter to be 1500 μm).[11,12] Drusen were defined as small drusen when their diameter were ≤ half the diameter of the vein (i.e. ≤ 63 μm), as large drusen for ≥ full diameter of the vein (i.e. ≥ 125 μm), and as intermediate drusen if anything in between (> 63 μm and < 125 μm). For borderline findings, the K-DRS image analysis software semi-automatically facilitated the measurement of drusen diameter, when the two distant points of the smallest drusen diameter were manually clicked by the grader. To assess total drusen area, the K-DRS software allowed for digitally placing a circle with 650 μm in diameter on the image, which helped the grader to categorize total drusen area as < or ≥ the circle area. GA was defined as an area of RPE atrophy ≥ a circle with 350 μm in diameter, central or paracentral localization, and the presence of at least two of the following features: sharply demarcated boarder, lack of RPE, visible choroidal vessels, and circular shape.[13] Pure GA was defined if central or paracentral GA, but no NV was present; pure NV was defined if NV, but no GA was present; mixed GA/NV was defined if both, GA and NV were detected.

Supplementary Table 2. Applied classification systems for AMD – the Clinical Classification. Shown are classification steps and detailed descriptions based on the Clinical Classification after Ferris et al., 2013 [11].

AMD classification[a,b]	Description
No AMD classification	Due to missing image, not gradable image (insufficient quality and/or obscuring lesions and/or competing retinal disease)
No AMD, no apparent aging changes	No drusen, no AMD-related pigmentary abnormalities[c]
No AMD, normal aging changes	Only (one or more) small Drusen ≤ 63 µm[d], no AMD-related pigmentary abnormalities[c]
Early AMD	Only (one or more) intermediate Drusen > 63 µm ≤ 125 µm, no AMD-related pigmentary abnormalities[c]
Intermediate AMD	(One or more) large Drusen > 125 µm and/or AMD-related pigmentary abnormalities[c]
Late AMD	Any geographic atrophy[e] and/or scaring/neovascular[f] AMD

Abbreviations: AMD = age-related macular degeneration;

[a]) Classification per person; the more severe eye was used to classify the person; if only one eye was gradable, this available eye was used to classify the person.

[b]) Only considering fundus lesions within 2 standard disc diameters (approx. 3000 µm) of the center of the macula/fovea.

[c]) Any definite hyper- or hypopigmentary abnormalities associated with medium or large drusen but not associated with known retinal disease entities or other reasons for such abnormalities.

[d]) For determination of drusen size, the shortest drusen diameter was compared to that of an average normal retinal vein at the disc margin, considered to be approximately one-twelfth disc diameter or approximately 125 µm, when the average disc diameter is taken as 1500 µm.[12]

[e]) Area of atrophy ≥ circle with 350 µm in diameter, central or paracentral localization, presence of at least two of these features: sharp edge, lack of RPE, visible choroidal vessels, circular shape.[13] Lesions with an area < circle with 350 µm in diameter were graded as pigmentary abnormalities.

[f]) Presence of any of the following: pigment epithelial and/or retinal detachment, subretinal hemorrhage, scaring, subretinal new vessels.

Supplementary Table 3. Applied classification systems for AMD – the Three Continent AMD Consortium Severity Scale. Shown are classification steps and detailed descriptions based on the Three Continent AMD Consortium Severity Scale after Klein et al., 2014 [13].

AMD classification[a,b]	Description
No AMD classification	Due to missing image, not gradable image (insufficient quality and/or obscuring lesions and/or competing retinal disease)
No AMD	No drusen or only (one or more) small-intermediate drusen (< 125 µm[c]), no AMD-related pigmentary abnormalities[d]<br>No drusen, only any pigmentary abnormalities
Mild early AMD	(One or more) small-intermediate drusen (< 125 µm), and AMD-related pigmentary abnormalities[d]<br>(One or more) large Drusen with drusen area < O2 circle[e], no AMD-related pigmentary abnormalities[d]
Moderate early AMD	(One or more) large Drusen with drusen area < O2 circle[e] and AMD-related pigmentary abnormalities[d]<br>(One or more) large Drusen with drusen area ≥ O2 circle[e], no AMD-related pigmentary abnormalities[d]
Severe early AMD	(One or more) large Drusen with drusen area ≥ O2 circle[e], and AMD-related pigmentary abnormalities[d]
Late AMD	Any geographic atrophy[f], no scaring/neovascular[g] AMD<br>Scaring/neovascular[g] AMD and/or any geographic atrophy[f]

Abbreviations: AMD = age-related macular degeneration; O2 = ”outer”, standard circle as defined by the Three Continent AMD Consortium [13];

[a]) Classification per person; the more severe eye was used to classify the person; if only one eye was gradable, this available eye was used to classify the person.

[b]) Only considering fundus lesions within 2 standard disc diameters (approx. 3000 µm) of the center of the macula/fovea.

[c]) For determination of drusen size, the shortest drusen diameter was compared to that of an average normal retinal vein at the disc margin, considered to be approximately one-twelfth disc diameter or approximately 125 µm, when the average disc diameter is taken as 1500 µm.[12]

[d]) Any definite hyper- or hypopigmentary abnormalities associated with medium or large drusen but not associated with known retinal disease entities or other reasons for such abnormalities.

[e]) O2 circle is defined as a circle with diameter of 650 µm; drusen area equivalent to O2 circle accounts to 331.820 $µm^2$.

[f]) Area of atrophy ≥ circle with 350 µm in diameter, central or paracentral localization, presence of at least two of these features: sharp edge, lack of RPE, visible choroidal vessels, circular shape.[13] Lesions with an area < circle with 350 µm in diameter were graded as pigmentary abnormalities.

[g]) Presence of any of the following: pigment epithelial and/or retinal detachment, subretinal haemorrhage, scaring, subretinal new vessels.

Supplementary Text 4. Correcting AMD prevalence estimates for the bias from participants with only one gradable eye.

The standard procedure for assessing AMD disease status of a participant is to analyze each eye and utilize the result of the worse eye to define the AMD status of the participant. However, in almost all epidemiological studies, there are usually participants with a gradable image only available for one eye and the question arises whether these can be utilized for AMD grading and AMD prevalence estimation.

In our study with $n = 1040$ analyzed participants, $n_f = 155$ participants were classified based on only one eye (*one-eye participants*), the other $n_v = 885$ on both (*two-eye participants*). While we here assume an AMD classification system with five categories where category one classifies the AMD-free participants and higher categories denote a more severe disease status, the here presented approach can be readily extended to grading systems with more or fewer categories. Utilizing the observed disease status of a sole eye as disease stage for a one-eye participant will yield a less severe stage for the participant, when the less affected eye was the sole observed eye. We assume for the following that the missing process is random, i.e. independent of the disease status of the eye. Thus, the prevalence estimates based on the observed disease status of sole eyes of one-eye participants are biased in favor of lower disease categories. In the following, we present an approach to estimate the true prevalence for each AMD status $\mathrm{k} \in \{1, \ldots, 5\}$ based on two- and one-eye participants that adjusts for the misclassification in one-eye participants.

Let $Y_i \in \{1, \ldots, 5\}$ be the true (potentially unobserved) AMD status of a participant i (i.e. AMD status of the worse eye) and $Y_i^* \in \{1, \ldots, 5\}$ the observed AMD status of one (randomly selected) eye. Thus, for the two-eye participants, observations on Y and Y^* are available, for the one-eye participants, only observations on Y^*. If we additionally assume that the n_v two-eye participants are a random subsample of the n participants, they represent an *internal validation sample* [1]. In this case the observed disease stage relative frequencies for two-eye participants are valid unbiased estimates of the AMD prevalence in the overall population. An appropriate additional consideration of the n_f potentially misclassified participants can,

however, yield an unbiased estimate with smaller standard error compared to the scenario where only the two-eye participants are utilized.

We can describe the misclassification procedure in the one-eye participants by *predictive values*: For a five-category AMD status, let $\Lambda(Y|Y^*)$ be the 5×5 matrix of predictive values with entries $\lambda_{\mathrm{kl}} = P(\mathrm{Y} = \mathrm{k}|\mathrm{Y}^* = \mathrm{l}),\ \mathrm{k}, \mathrm{l} \in \{1, \dots, 5\}$, which denote the probabilities that the persons' true AMD stage is k (worse eye) given that the stage observed in one eye is l. Since the true AMD stage (worse eye) is always higher than or equal to the AMD stage observed in one eye, all entries above the main diagonal of this matrix are zero.

Using the law of total probability, it is in general possible to rewrite the probability of the true AMD stage k, $P(Y = k)$, in terms of the predictive values and the probabilities of the error-prone observed AMD stage l, $P(Y^* = l)$, for $k, l = 1, \dots, 5$, as

$$P(Y = k) = \sum_{l=1}^{5} P(\mathrm{Y} = \mathrm{k}|\mathrm{Y}^* = \mathrm{l}) \times P(Y^* = l). \qquad (1)$$

Using a matrix notation, this can be expressed for all categories through

$$P_Y = \Lambda(Y|Y^*) \times P_{Y^*}, \qquad (2)$$

with the vectors $P_Y = \big(P(Y = 1), \dots, P(Y = 5)\big)'$ and $P_{Y^*} = \big(P(Y^* = 1), \dots, P(Y^* = 5)\big)'$.

The estimate of the probability for the error-prone observed AMD stage k is given by the observed relative frequency of AMD stage k among the one-eye participants, $\hat{P}^f(Y^* = k)$. To derive estimates of the predictive values, $\hat{\lambda}_{\mathrm{kl}}$, $k, l = 1, \dots, 5$, we can utilize the internal validation sample of two-eye participants, as we observe both Y and Y^* for these individuals: The column l of the matrix of predictive values, $\Lambda(Y|Y^*)_{\cdot l} = (\lambda_{1l}, \dots, \lambda_{5l})'$, represents the distribution of true (worse eye) disease stages in participants with at least one eye graded in disease stage l. This is exactly how we estimate the predictive values: For each $l = 1, \dots, 5$, we compute the relative frequencies of worse eye classifications Y of all two-eye participants with at least one single eye classified in disease stage l.

To derive a bias-corrected AMD stage k prevalence among one-eye participants, we add the observed AMD stage l relative frequency among one-eye participants, $\hat{P}^f(Y^* = l)$, multiplied with the estimated predictive values, $\hat{\lambda}_{\mathrm{kl}}$, across $\mathrm{l} \in \{1, \dots, 5\}$ in analogy to (2) as $\sum_{l=1}^{5} \hat{P}(\mathrm{Y} = \mathrm{k} | \mathrm{Y}^* = \mathrm{l}) \times \hat{P}^f(Y^* = l)$. This estimate combined with the observed AMD stage k prevalence among two-eye participants, $\hat{P}^v(Y = k)$, weighted by proportion of one-eye participants, $\frac{n_f}{n}$, and the proportion of two-eye participants, $\frac{n_v}{n}$, respectively, yield a bias-corrected estimate of the true disease stage k probability on all (one- and two-eye) observations,

$$\hat{P}(Y = k) = \frac{n_v}{n} \hat{P}^v(Y = k) + \frac{n_f}{n} \sum_{l=1}^{5} \hat{P}(\mathrm{Y} = \mathrm{k} | \mathrm{Y}^* = \mathrm{l}) \times \hat{P}^f(Y^* = l). \tag{3}$$

Using a matrix notation, this can be expressed as

$$\hat{P}_Y = \frac{n_v}{n} \hat{P}_Y^v + \frac{n_f}{n} \hat{\Lambda}\,(Y | Y^*) \times \hat{P}_{Y^*}^f\,. \tag{4}$$

with the vectors $\hat{P}_Y$ denoting the bias-corrected overall prevalence estimates (disease stage probabilities), $\hat{P}_Y^v$ denoting the disease stage probability estimates for the two-eye participants, and $\hat{P}_{Y^*}^f$ (as above) denoting the biased disease stage probability estimate of the one-eye participants. This approach to correct for misclassification is often referred to as *adjustment using predictive values* or *adjustment using calibration probabilities*.[14,15]

Tenenbein (1972) [16] shows that (4) is the maximum likelihood estimate of the true class probabilities under misclassification with an internal validation sample (assuming a multinomial distribution of true class counts) and derives formulas to calculate asymptotic variances of the estimates using the delta method. Following maximum likelihood theory, the estimates are therefore asymptotically efficient. Kuha and Skinner (1997) [14] compare this approach to the adjustment using the misclassification probabilities $\Theta(Y^* | Y)$ (*matrix method*) and show in an example that the latter is, in situations where the adjustment using predictive values is adequate, less efficient.

To obtain bias-corrected AMD prevalence estimates standardized to the Bavarian population, the bias-adjusted disease stage probabilities for each sex and 5-year age-group can be estimated by (4) and then combined by a weighted sum, with weights corresponding to the proportion of the respective groups in the Bavarian population. In general, it would be possible to estimate different predictive values for each age-sex group, which would be important if the predictive values differed by age-sex group. However due to only few (two-eye) observations for some of the age-sex groups, these estimates turn out to be rather unstable. Therefore we decided to assume common predictive values for all age-sex groups and estimate them based on all two-eye observations.

Variance estimates for the standardized prevalence estimates could again be derived asymptotically using the delta method, as an alternative we propose to use a non-parametric bootstrap procedure.

Supplementary Table 4. Characteristics of recruited and analyzed subjects from the AugUR study. Shown are the distribution of age, sex, lifestyle factors, metabolic parameters and self-reported eye diseases/conditions for all participants (n = 1,133) and separately for those without acquired fundus images, without any eye gradable, and for those constituting the analyzed sample (at least one eye gradable).

	**All participants (n=1,133)**	**No image for any eye acquired (n=4)**	**Image for at least one eye acquired (n=1,129)**		**P-value for difference**	
			**No eye gradable (n=89)**	**At least one eye gradable (n=1,040)**	**At least one eye gradable (n=1,040) vs. No image for any eye acquired + No eye gradable (n=93)**	
					**unadjusted**	**adjusted[a]**
**General characteristics, lifestyle factors and metabolic parameters**						
Age [years], mean ± SD	77.6 ± 5.0	79.2 ± 4.4	79.0 ± 4.9	77.5 ± 5.1	**$2.6*10^{-3}$**	**0.01**
Men, % (n)	54.9 (622)	50.0 (2)	62.9 (56)	54.2 (564)	0.13	0.17
Current smoker[b], % (n)	6.5 (74)	0.0 (0)	11.2 (10)	6.2 (64)	0.09	0.07
Ex-smoker[b], % (n)	38.0 (431)	25.0 (1)	41.6 (37)	37.8 (393)	0.15	0.26
Pack years[c], mean ± SD	27.5 ± 30.2	0	23.9 ± 19.7	27.8 ± 31.0	0.79	0.50
BMI[d] [kg m^{-2}], mean ± SD	28.0 ± 4.5	25.1 ± 6.8	28.2 ± 4.5	28.0 ± 4.5	0.78	0.68
T2DM[e], % (n)	21.5 (244)	50.0 (2)	29.2 (26)	20.8 (216)	**0.04**	**0.05**
Hypertension[f], % (n)	73.5 (830)	100.0 (4)	79.8 (71)	72.8 (744)	0.10	0.11
**Eye-related parameters**						
Cataract[g], % (n)	49.0 (555)	25.0 (1)	53.9 (48)	48.7 (506)	**$2.6*10^{-3}$**	0.90
Cataract surgery[h], % (n)	69.2 (384)	0.0 (0)	68.8 (33)	69.4 (351)	0.77	0.50
Glaucoma[g], % (n)	7.3 (83)	0.0 (0)	10.1 (9)	7.1 (74)	0.06	0.60
Diabetic retinopathy[g], % (n)	1.2 (14)	0.0 (0)	3.4 (3)	1.1 (11)	**$7.0*10^{-4}$**	0.44
Pupil size[i] [mm], mean ± SD	3.6 ± 0.7	NA	2.7 ± 0.7	3.7 ± 0.7	**$2.9*10^{-19}$**	**$3.3*10^{-23}$**
Pharmacological mydriasis, % (n)	61.6 (698)	NA	48.3 (43)	63.0 (655)	**0.03**	**0.01**
Pupil size[i] without mydriasis [mm], mean ± SD	3.2 ± 0.5	NA	2.6 ± 0.4	3.3 ± 0.5	**$3.5*10^{-14}$**	**$1.7*10^{-10}$**
Pupil size[i] with mydriasis [mm], mean ± SD	3.8 ± 0.7	NA	3.0 ± 0.9	3.9 ± 0.6	**$4.6*10^{-11}$**	**$3.1*10^{-13}$**

Bold values indicate significant P-values.
Abbreviations: SD = standard deviation; BMI = body-mass-index; T2DM = type 2 diabetes; NA = not available;
[a]) Adjusted P: age- and sex-adjusted, except for “Age” (only sex-adjusted). Adjustment for age^2 did not alter results.
[b]) Currently smoking ≥ 1 cigarette per month; having stopped smoking for ≥ 1 month.
[c]) Pack years are defined as number of packs (20 cigarettes per pack) smoked per day times the number of years of smoking, estimating that the participant has started smoking at the age of 18 years.
[d]) BMI is defined as measured weight divided by squared measured body height.
[e]) T2DM is defined as a self-reported diagnosis or anti-diabetes medication intake.
[f]) Hypertension is defined as actually measured systolic blood pressure of ≥ 140 mmHg, diastolic blood pressure of ≥ 90 mmHg or corresponding medication taken, given that the participants were aware of having hypertension.
[g]) History of cataract, glaucoma and diabetic retinopathy was assessed via self-report.
[h]) History of cataract surgery was assessed via self-report among those with reported cataract.
[i]) Pupil size per person is defined as the smaller pupil diameter of both eyes.

Supplementary Table 5. Observed relative frequencies of AMD status for two classification systems by sex and five-year age-groups. Shown are the observed frequencies (for men and women in parentheses) for each AMD status based on the Clinical Classification [11] and the Three Continent AMD Consortium Severity Scale [13] in the 1,040 analyzed individuals with at least one eye gradable.

	**70-74 years**	**75-79 years**	**80-84 years**	**85-89 years**	**90-95 years**	**All**
N in study (men / women)	375 (190 / 185)	380 (208 / 172)	181 (102 / 79)	80 (51 / 29)	24 (13 / 11)	1040 (564 / 476)
**Clinical Classification**						
No AMD, no apparent aging changes, %	28.8 (31.1 / 26.5)	30.8 (31.3 / 30.2)	26.0 (29.4 / 21.5)	16.3 (19.6 / 10.3)	4.2 (0.0 / 9.1)	27.5 (29.1 / 25.6)
No AMD, normal aging changes, %	28.0 (29.0 / 27.0)	21.1 (24.5 / 16.9)	17.1' (19.6 / 13.9)	22.5 (21.6 / 24.1)	20.8 (23.1 / 18.2)	23.0 (24.8 / 20.8)
Early AMD, %	25.1 (21.6 / 28.7)	27.9 (24.0 / 32.6)	26.5 (21.6 / 32.9)	22.5 (19.6 / 27.6)	37.5 (30.8 / 45.5)	26.4 (22.5 / 31.1)
Intermediate AMD, %	16.0 (16.3 / 15.7)	17.4 (16.4 / 18.6)	17.1 (17.7 / 16.5)	20.0 (21.6 / 17.2)	16.7 (23.1 / 9.1)	17.0 (17.2 / 16.8)
Late AMD, %	2.1 (2.1 / 2.2)	2.9 (3.9 / 1.7)	13.3 (11.8 / 15.2)	18.8 (17.7 / 20.7)	20.8 (23.1 / 18.2)	6.1 (6.4 / 5.7)
**Three Continent AMD Consortium Severity Scale**						
No AMD, %	81.9 (81.6 / 82.2)	80.3 (79.8 / 80.8)	69.6 (70.6 / 68.4)	61.3 (60.8 / 62.1)	62.5 (53.9 / 72.7)	77.1 (76.4 / 77.9)
Mild early AMD, %	9.9 (12.1 / 7.6)	8.4 (9.1 / 7.6)	8.8 (8.8 / 8.9)	8.8 (7.8 / 10.3)	4.2 (7.7 / 0.0)	8.9 (9.9 / 7.8)
Moderate early AMD, %	3.5 (2.1 / 4.9)	4.5 (2.4 / 7.0)	5.0 (4.9 / 5.1)	5.0 (5.9 / 3.5)	4.2 (7.7 / 0.0)	4.2 (3.2 / 5.5)
Severe early AMD, %	2.7 (2.1 / 3.2)	4.0 (4.8 / 2.9)	3.3 (3.9 / 2.5)	6.3 (7.8 / 3.5)	8.3 (7.7 / 9.1)	3.7 (4.1 / 3.2)
Late AMD, %	2.1 (2.1 / 2.2)	2.9 (3.9 / 1.7)	13.3 (11.8 / 15.2)	18.8 (17.7 / 20.7)	20.8 (23.1 / 18.2)	6.1 (6.4 / 5.7)

Abbreviations: AMD = age-related macular degeneration;

Supplementary Table 6. Prevalence estimates of AMD for two classification systems by five-year age-groups. Shown are prevalence estimates age- and sex-standardized to the Bavarian population, for each AMD status based on the Clinical Classification [11] and the Three Continent AMD Consortium Severity Scale [13] in the 1,040 analyzed individuals with at least one eye gradable.

	**70-74 years**	**75-79 years**	**80-84 years**	**85-89 years**	**90-95 years**	**All**
N in Bavarian population (men / women)[a]	706551 (328967 / 377584)	467451 (202087 / 265364)	340209 (130709 / 209500)	202497 (57207 / 145290)	69922 (16148 / 53774)	1786630 (735118 / 1051512)
**Clinical Classification**						
No AMD, no apparent aging changes, %	28.6	30.7	24.6	13.0	7.0	25.8 (29.2 / 23.3)
No AMD, normal aging changes, %	27.9	20.2	16.1	23.4	19.3	22.8 (25.4 / 21.0)
Early AMD, %	25.4	28.9	28.6	25.3	42.1	27.5 (22.3 / 31.2)
Intermediate AMD, %	16.0	17.6	16.9	18.5	12.3	16.7 (17.1 / 16.5)
Late AMD, %	2.1	2.7	13.9	19.8	19.3	7.2 (6.0 / 8.0)
**Three Continent AMD Consortium Severity Scale**						
No AMD, %	81.9	80.4	69.2	61.7	68.4	76.3 (76.9 / 75.8)
Mild early AMD, %	9.7	8.2	8.9	9.6	1.8	8.8 (10.3 / 7.8)
Moderate early AMD, %	3.6	5.0	5.0	4.1	1.8	4.2 (3.1 / 5.0)
Severe early AMD, %	2.7	3.7	3.1	4.7	8.8	3.5 (3.7 / 3.3)
Late AMD, %	2.1	2.7	13.9	19.8	19.3	7.2 (6.0 / 8.0)

Abbreviations: AMD = age-related macular degeneration;
[a]) Numbers derived from https://www.statistik.bayern.de/statistik/zensus/00843.php.

Supplementary Table 7. Frequencies of specific AMD features observed per eye in the AugUR study. Shown are AMD features per eye for 1,040 participants with images for at least one eye acquired, separated for right and left eyes.

	70-74 years		75-79 years		80-84 years		85-89 years		90-95 years		All	
	**OD**	**OS**	**OD**	**OS**	**OD**	**OS**	**OD**	**OS**	**OD**	**OS**	**OD**	**OS**
	n=349	n=357	n=350	n=352	n=164	n=165	n=70	n=73	n=22	n=23	n=955	n=970
**Drusen**												
Small Drusen, n (%)	84 (21.1)	95 (23.8)	72 (17.6)	68 (16.6)	28 (13.6)	32 (15.5)	12 (13.5)	13 (14.6)	6 (23.1)	2 (7.7)	202 (17.9)	210 (18.6)
Intermediate Drusen, n (%)	90 (22.6)	89 (22.3)	84 (20.5)	101 (24.7)	40 (19.4)	44 (21.4)	17 (19.1)	20 (22.5)	5 (19.2)	8 (30.8)	236 (20.9)	262 (23.2)
Large Drusen, n (%)	35 (8.8)	36 (9.0)	41 (10.0)	43 (10.5)	28 (13.6)	20 (9.7)	9 (10.1)	11 (12.4)	3 (11.5)	4 (15.4)	116 (10.3)	114 (10.1)
Large Drusen with overall drusen area < O2 circle[a], n (%)	21 (5.3)	22 (5.5)	13 (3.2)	21 (5.1)	16 (7.8)	8 (3.9)	4 (4.5)	4 (4.5)	0 (0.0)	2 (7.7)	54 (4.8)	57 (5.1)
Large Drusen with overall drusen area ≥ O2 circle[a], n (%)	14 (3.5)	14 (3.5)	28 (6.9)	22 (5.4)	12 (5.8)	12 (5.8)	5 (5.6)	7 (7.9)	3 (11.5)	2 (7.7)	62 (5.5)	57 (5.1)
**Pigmentary abnormalities**												
Pigment abnormalities not related to AMD, n (%)	19 (4.8)	23 (5.8)	29 (7.1)	32 (7.8)	15 (7.3)	10 (4.9)	5 (5.6)	6 (6.7)	3 (11.5)	3 (11.5)	71 (6.3)	74 (6.6)
Pigment abnormalities related to AMD[b], n (%)	16 (4.0)	19 (4.8)	26 (6.4)	19 (4.8)	16 (7.8)	15 (7.3)	8 (9.0)	11 (12.4)	2 (7.7)	3 (11.5)	68 (6.0)	65 (5.8)
**Geographic atrophy[c]**												
Paracentral GA without scaring / neovascular AMD, n (%)	1 (0.3)	1 (0.3)	0 (0.0)	0 (0.0)	2 (1.0)	5 (2.4)	1 (1.1)	2 (2.3)	0 (0.0)	0 (0.0)	4 (0.4)	8 (0.7)
Central[d] GA without scaring / neovascular AMD, n (%)	0 (0.0)	0 (0.0)	1 (0.2)	0 (0.0)	2 (1.0)	2 (1.0)	3 (3.4)	3 (3.4)	3 (11.5)	3 (11.5)	9 (0.8)	8 (0.7)
**Scaring / neovascular AMD[e]**												
Scaring / neovascular AMD without any form of GA, n (%)	1 (0.3)	5 (1.3)	3 (0.7)	4 (1.0)	11 (5.3)	10 (4.9)	5 (5.6)	4 (4.5)	1 (3.9)	1 (3.9)	21 (1.9)	24 (2.1)
Scaring / neovascular AMD with any form of GA, n (%)	2 (0.5)	2 (0.5)	3 (0.7)	3 (0.7)	2 (1.0)	1 (0.5)	2 (2.3)	3 (3.4)	0 (0.0)	0 (0.0)	9 (0.8)	9 (0.8)

Abbreviations: OD = right eye; OS = left eye; O2 = ”outer”, standard circle as defined by the Three Continent AMD Consortium [13]; GA = geographic atrophy;

[a]) O2 circle is defined as a circle with diameter of 650 µm; drusen area equivalent to O2 circle accounts to 331.820 $µm^2$.
[b]) Any definite hyper- or hypopigmentary abnormalities associated with medium or large drusen but not associated with known retinal disease entities or other reasons for such abnormalities.
[c]) Area of atrophy ≥ 0.5 disc area, central or paracentral localization, presence of at least two of these features: sharp edge, lack of RPE, visible choroidal vessels, circular shape.
[d]) Area of GA affecting area of fixation.
[e]) Presence of any of the following: pigment epithelial and/or retinal detachment, subretinal haemorrhage, scaring, subretinal new vessels.

Supplementary Text 5. AMD features per eye.

When evaluating specific AMD features per eye (drusen, pigmentary abnormalities, details on GA or NV), we found the following (**Supplementary Table 7**): (i) there were no obvious differences of any AMD features between right and left eyes. (ii) Approximately 50% of pigment abnormalities were "unrelated to AMD", as defined by Ferris et al. [11] (no co-incidence with medium or large drusen). Such "pigmentary changes unrelated to AMD" are thought to be linked to other retinal disease entities or other reasons for such abnormalities (e.g. myopic fundus alterations, central serous chorioretinopathy, status post inflammation, status post laser treatment or retinal surgery, adult vitelliform macular dystrophy, or other hereditary retinal dystrophies). According to the definition by Ferris and colleagues, such features were not considered as any type of AMD. Interestingly, the proportion of "pigmentary changes unrelated to AMD" was quite high (6.3/6.6% right eye/left eye) and increased by age in a similar fashion as the proportion of AMD-related changes. (iii) We observed a decreasing trend by age for small drusen relative frequencies (21.1/23.8% to 13.5/14.6% comparing those aged 70-74 with those at 85-89), no age trend for intermediate drusen, and an increasing trend by age for large drusen (from 8.8/9.0% to 10.1/12.4%).

Supplementary Table 8. Concordance of AMD status between two graders applying the Clinical Classification. Shown are cross tabulations of AMD stages as derived by each of the two graders for the Clinical Classification [11]. From the 450 participants included in this double grading exercise, 422 had gradable images for at least one eye and are analyzed here.

		Grader 2 (TB)					
		No AMD, no apparent aging changes, n	No AMD, normal aging changes, n	Early AMD, n	Intermediate AMD, n	Late AMD, n	**Total, n**
**Grader 1 (CB)**	No AMD, no apparent aging changes, n	47	43	1	1	0	92
	No AMD, normal aging changes, n	0	105	0	2	0	107
	Early AMD, n	0	10	114	4	0	128
	Intermediate AMD, n	1	3	0	67	0	71
	Late AMD, n	0	0	0	3	21	24
**Total, n**		48	161	115	77	21	422

Abbreviations: AMD = age-related macular degeneration;

Supplementary Table 9. Concordance of AMD status between two graders applying the Three Continent AMD Consortium Severity Scale. Shown are cross tabulations of AMD stages as derived by each of the two graders for the Three Continent AMD Consortium Severity Scale [13]. From the 450 participants included in this double grading exercise, 422 had gradable images for at least one eye and are analyzed here.

		Grader 2 (TB)					
		No AMD, n	Mild early AMD, n	Moderate early AMD, n	Severe early AMD, n	Late AMD, n	**Total, n**
**Grader 1 (CB)**	No AMD, n	320	8	0	0	0	328
	Mild early AMD, n	5	29	3	0	0	37
	Moderate early AMD, n	0	1	18	0	0	19
	Severe early AMD, n	0	0	0	14	0	14
	Late AMD, n	0	1	0	2	21	24
**Total, n**		325	39	21	16	21	422

Abbreviations: AMD = age-related macular degeneration;

Supplementary Table 10. Evaluation of the effect of single eye grading based on the 885 participants with both eyes gradable in a five-category interpretation. Shown are AMD stages utilizing the worst of the two gradable eyes, only the right, the left, or a random eye based on the Clinical Classification [11] and the Three Continent AMD Consortium Severity Scale [13] for the five-category scale.

	**Worst eye graded**	**Only OD graded**	**Only OS graded**	**Random eye graded**
**Clinical Classification**				
No AMD, no apparent aging changes, %	25.6	37.2	34.2	36.5
No AMD, normal aging changes, %	22.9	20.7	21.8	20.8
Early AMD, %	26.9	23.2	24.1	23.2
Intermediate AMD, %	18.2	14.4	14.9	14.5
Late AMD, %	6.3	4.6	5.0	5.1
**Three Continent AMD Consortium Severity Scale**				
No AMD, %	75.7	81.2	80.1	80.5
Mild early AMD, %	9.3	6.2	7.5	7.1
Moderate early AMD, %	4.6	3.7	4.4	4.1
Severe early AMD, %	4.1	4.2	3.1	3.3
Late AMD, %	6.3	4.6	5.0	5.1

Abbreviations: OD = right eye; OS = left eye; AMD = age-related macular degeneration;

Supplementary Table 11. Evaluation of the effect of single eye grading based on the 885 participants with both eyes gradable in a three-category interpretation. Shown are AMD stages utilizing the worst of the two gradable eyes, only the right, the left, or a random eye based on the Clinical Classification [11] and the Three Continent AMD Consortium Severity Scale [13] for the three categories “no AMD”, “any early or intermediate AMD”, and ”late AMD”.

	Worst eye graded	Only OD graded	Only OS graded	Random eye graded
**Clinical Classification**				
No AMD, %	48.6	57.9	56.0	57.3
“Any early or “intermediate” AMD[a], %	45.1	37.5	39.0	37.7
Late AMD, %	6.3	4.6	5.0	5.1
**Three Continent AMD Consortium Severity Scale**				
No AMD, %	75.7	81.2	80.1	80.5
“Any early” AMD[b], %	18.0	14.1	14.9	14.5
Late AMD, %	6.3	4.6	5.0	5.1

Abbreviations: OD = right eye; OS = left eye; AMD = age-related macular degeneration;
[a]) For the Clinical Classification collapsing early AMD and intermediate AMD.
[b]) For the Three Continent AMD Consortium Severity Scale, collapsing mild early AMD, moderate early AMD, and severe early AMD to “any early” AMD.

Supplementary Table 12. Predictive Values for the five-category scale. Shown are the estimated predictive values estimated based on the 855 participants with both gradable eyes using the Clinical Classification [11] and the Three-Continent AMD Consortium Severity Scale [13] for the five-category scale. These predictive values are computed based on the two-eye participants and used for the bias-correction of the relative AMD frequencies for the one-eye observations. The true AMD stage (defined as worst eye AMD stage) and the observed AMD stage (one eye) are denoted by Y and Y*, respectively.

Clinical Classification

Y \ Y*	No AMD, no apparent aging	No AMD, normal aging	Early AMD	Inter-mediate AMD	Late AMD
No AMD, no apparent aging	0.718	0.000	0.000	0.000	0.000
No AMD, normal aging	0.172	0.790	0.000	0.000	0.000
Early AMD	0.084	0.168	0.861	0.000	0.000
Inter-mediate AMD	0.025	0.040	0.124	0.923	0.000
Late AMD	0.000	0.003	0.014	0.077	1.000

Three Continent AMD Consortium Severity Scale

Y \ Y*	No AMD	Mild early AMD	Mode rate early AMD	Severe early AMD	Late AMD
No AMD	0.938	0.000	0.000	0.000	0.000
Mild early AMD	0.046	0.818	0.000	0.000	0.000
Mode rate early AMD	0.008	0.116	0.778	0.000	0.000
Severe early AMD	0.003	0.033	0.153	0.828	0.000
Late AMD	0.005	0.033	0.069	0.172	1.000

Abbreviations: AMD = age-related macular degeneration;

Supplementary Table 13. Predictive Values for the three-category scale. Shown are the estimated predictive values estimated based on the 855 participants with both gradable eyes using the Clinical Classification [11] and the Three-Continent AMD Consortium Severity Scale [13] for the three categories “no AMD”, “any early or intermediate AMD”, and ”late AMD”. These predictive values are computed based on the two-eye participants and used for the bias-correction of the relative AMD frequencies for the one-eye observations. The true AMD stage (defined as worst eye AMD stage) and the observed AMD stage (one eye) are denoted by Y and Y*, respectively.

Clinical Classification

Y \ Y*	No AMD	“Any early/ intermediate” AMD	Late AMD
No AMD	0.853	0.000	0.000
“Any early/inter-mediate” AMD[a]	0.146	0.962	0.000
Late AMD	0.001	0.038	1.000

Three Continent AMD Consortium Severity Scale

Y \ Y*	No AMD	“Any early” AMD	Late AMD
No AMD	0.938	0.000	0.000
“Any early” AMD[b]	0.057	0.922	0.000
Late AMD	0.005	0.078	1.000

Abbreviations: AMD = age-related macular degeneration;
[a]) For the Clinical Classification collapsing early AMD and intermediate AMD.
[b]) For the Three Continent AMD Consortium Severity Scale, collapsing mild early AMD, moderate early AMD, and severe early AMD to “any early” AMD.

Supplementary Table 14. Bias-corrected observed relative frequencies for two classification systems by sex and five-year age-groups. Shown are the bias-corrected observed frequencies for each AMD status based on the Clinical Classification [11] and the Three Continent AMD Consortium Severity Scale [13] in the 1,040 analyzed individuals.

	**70-74 years (n=375)**		**75-79 years (n=380)**		**80-84 years (n=181)**		**85-89 years (n=80)**		**90-95 years (n=42)**		**All (n=1040)**
	Men	Women	Men	Women	Men	Women	Men	Women	Men	Women	
N in study	190	185	208	172	102	79	51	29	13	11	
**Clinical Classification**											
No AMD, no apparent aging changes, %	30.2	24.4	29.5	28.9	26.4	19.7	18.5	10.3	0.0	9.1	25.9
No AMD, normal aging changes, %	28.8	28.0	24.3	17.2	20.4	14.7	21.0	23.4	23.1	18.2	23.2
Early AMD, %	21.9	29.3	25.1	32.9	22.9	33.0	20.4	27.2	28.6	44.2	27.0
Intermediate AMD, %	16.8	16.2	17.1	19.2	18.3	17.2	21.9	18.2	25.0	10.2	17.6
Late AMD, %	2.3	2.2	4.0	1.9	12.0	15.4	18.2	20.8	23.3	18.3	6.2
**Three Continent AMD Consortium Severity Scale**											
No AMD, %	81.0	81.5	78.8	80.2	69.4	67.6	59.9	61.4	61.4	72.2	76.3
Mild early AMD, %	12.2	8.1	9.8	7.9	9.3	9.2	7.8	10.8	10.8	0.4	9.3
Moderate early AMD, %	2.4	4.8	2.6	7.0	5.1	5.3	6.5	3.5	3.5	0.1	4.4
Severe early AMD, %	2.2	3.4	4.8	3.0	4.2	2.6	7.7	3.5	3.5	9.1	3.7
Late AMD, %	2.2	2.3	4.0	1.9	12.0	15.3	18.2	20.7	20.7	18.2	6.2

Abbreviations: AMD = age-related macular degeneration;

Supplementary Table 15. Bias-corrected prevalence estimates for two classification systems by five-year age-groups. Shown are the bias-corrected prevalence estimates age- and sex-standardized to the Bavarian population, for each AMD status based on the Clinical Classification [11] and the Three Continent AMD Consortium Severity Scale [13] in the 1,040 analyzed individuals.

	**70-74 years**	**75-79 years**	**80-84 years**	**85-89 years**	**90-95 years**	**Age-sex standardized prevalence [CI][a]**
N in Bavarian population (men / women)[b]	706551 (328967 / 377584)	467451 (202087 / 265364)	340209 (130709 / 209500)	202497 (57207 / 145290)	69922 (16148 / 53774)	1811231 (744435 / 1066796)
**Clinical Classification**						
No AMD, no apparent aging changes, %	27.1	29.2	22.3	12.6	7.0	24.3 [21.5; 27.1]
No AMD, normal aging changes, %	28.4	20.2	16.9	22.7	19.3	23.1 [20.2; 26.1]
Early AMD, %	25.8	29.5	29.1	25.3	40.6	27.9 [24.8; 31.1]
Intermediate AMD, %	16.4	18.3	17.6	19.3	13.6	17.4 [14.9; 19.9]
Late AMD, %	2.3	2.8	14.1	20.1	19.5	7.4 [5.4; 9.4]
**Three Continent AMD Consortium Severity Scale**						
No AMD, %	81.2	79.6	68.3	61.0	67.7	75.5 [72.5; 78.5]
Mild early AMD, %	10.0	8.7	9.2	10.0	2.3	9.2 [7.3; 11.2]
Moderate early AMD, %	3.7	5.1	5.2	4.4	1.9	4.4 [3.1; 5.8]
Severe early AMD, %	2.8	3.8	3.2	4.7	8.8	3.6 [2.4; 5.0]
Late AMD, %	2.2	2.8	14.0	20.0	19,4	7.3 [5.4; 9.4]

Abbreviations: AMD = age-related macular degeneration;
[a]) 95%- non-parametric percentile Bootstrap confidence intervals (Cis) based on 10000 replications.
[b]) Numbers derived from https://www.statistik.bayern.de/statistik/zensus/00843.php.

Supplementary Table 16. AMD prevalence estimates in participants 70+ in AugUR and previously published population-based studies. Shown are prevalence estimates for participants ≥ 70 years of age from population-based cross-sectional studies of European ancestry sorted by location and number of participants.

**Study name** (Location)	**# subjects (m/f)[a]**	**Age [years]**	**Response rate in complete study sample [%]**	**Response rate in age groups 70+ [%]**	**Classification system for AMD**	**Prevalence of early AMD [%]**	**Prevalence of late AMD (GA/NV) [%]**	**References**
**Germany**								
AugUR<br>(Regensburg)	375<br>380<br>181<br>80<br>24	70-74<br>75-79<br>80-84<br>85-89<br>90-95	20.1	20.1	Clinical Classification [11] / Three Continent AMD Consortium Severity Scale [13]	41.3 / 16.0<br>46.5 / 17.0<br>45.5 / 16.9<br>43.8 / 18.5<br>54.4 / 12.3	2.1<br>2.7<br>13.9<br>19.8<br>19.3	Present manuscript
Gutenberg Health Study (GHS)<br>(Mainz)	834	**65**-74	60.3	NA	Modified Rotterdam Study classification [17]	24.6	0.7	Korb et al., 2014 [18]
KORA S4 fundus sub-study<br>(Augsburg)	156	70-75	67.0	56.3	AREDS 9-step severity scale [19]	26.3	1.9	Brandl et al., 2016 [3]
**Other European countries**								
Age, Gene/Environment Susceptibility (AGES) Reykjavik Study<br>(Iceland)	1580<br>1532<br>1192<br>436	70-74<br>75-79<br>80-84<br>85+	70.9	NA	Modified WARMGS [20]	13.0<br>23.9<br>29.5<br>36.0	1.5<br>4.3<br>11.3<br>19.0	Jonasson et al., 2011 [21]; Harris et al., 2007 [22]
European Eye Study (EUREYE)<br>(Norway, Estonia, Northern Ireland, France, Italy, Greece, Spain)	1558<br>926<br>616	70-74<br>75-79<br>80+	45.3	NA[b]<br>38.3[b]	Rotterdam Study classification [17]	NA<br>NA<br>NA	2.0 m / 2.4 f<br>4.5 m / 3.4 f<br>7.0 m / 16.3 f	Augood et al., 2006 [23]
Rotterdam Study (RS)[c]<br>(Netherlands)	2257<br>1244<br>326	**65**-74<br>75-84<br>85-98	72.0	NA<br>NA<br>32.0	Rotterdam Study classification [17]	NA<br>NA<br>NA	0.8<br>3.7<br>11.0	Vingerling et al., 1995 [24]; Hofman et al., 2011 [25]; Mitchell et al., 1995 [26]

Supplementary Table 16 continued

Bridlington Eye Assessment Project (BEAP)<br>(England)	1069<br>808<br>533<br>183<br>33	70-74<br>75-79<br>80-84<br>85-89<br>≥90	56.0	NA	Modified Rotterdam Study classification [17]	55.9<br>59.4<br>61.5<br>55.2<br>63.6[d]	2.0<br>5.3<br>7.3<br>15.8<br>21.2	Wilde et al., 2017 [27]
The Irish Longitudinal study on Ageing (TILDA)<br>(Republic of Ireland)	1256<br>402	**65**-74<br>75+	61.6	NA	Modified International Classification and Grading System for AMD [28]	7.3<br>11.0	0.5<br>2.2	Akuffo et al., 2015 [29]
**Other Caucasian ancestry**								
Blue Mountains Eye Study (BMES)[c]<br>(Australia)	1209<br>653<br>135	**65**-74<br>75-84<br>85-97	87.9	NA<br>NA<br>75.0	WARMGS [20]	8.5<br>15.5<br>28.0	0.7<br>5.4<br>18.5	Mitchell et al., 1995 [26]
Beaver Dam Eye Study (BDES)[c]<br>(USA)	1249<br>717	**65**-74<br>75+	83.1	NA	WARMGS [20]	18.0<br>29.7	1.4<br>7.1	Klein et al., 1992 [30]

Abbreviations: m = male; f = female; NA = not available via literature search; WARMGS = Wisconsin age-related maculopathy grading system;
[a)] With gradable color fundus images for at least one eye.
[b]) The response rate in individuals aged 65-74 years is given as 50.0%. The response rate of 38.3% refers to individuals aged ≥75 years.
[c]) Given are prevalence estimates before the harmonization of AMD classification by Klein et al., 2014.[13]
[d]) Early AMD is defined as modified Rotterdam Study classification grades 1-2 ("early" AMD) and 3 ("intermediate" AMD).

References

1 Holzapfel, C. *et al.* Genes and lifestyle factors in obesity: results from 12,462 subjects from MONICA/KORA. *International journal of obesity* **34**, 1538-1545 (2010).

2 Stark, K. *et al.* The German AugUR study: study protocol of a prospective study to investigate chronic diseases in the elderly. *BMC geriatrics* **15**, 130 (2015).

3 Brandl, C. *et al.* Features of Age-Related Macular Degeneration in the General Adults and Their Dependency on Age, Sex, and Smoking: Results from the German KORA Study. *PloS one* **11**, e0167181 (2016).

4 Wichmann, H. E., Gieger, C., Illig, T. & Group, M. K. S. KORA-gen--resource for population genetics, controls and a broad spectrum of disease phenotypes. *Gesundheitswesen* **67 Suppl 1**, S26-30 (2005).

5 Meisinger, C. *et al.* Regional disparities of hypertension prevalence and management within Germany. *Journal of hypertension* **24**, 293-299 (2006).

6 Leitritz, M. A. *et al.* [Development and first results of fast and cost-effective examination methods for an ophthalmological screening within the National Cohort]. *Klinische Monatsblatter fur Augenheilkunde* **230**, 1238-1246 (2013).

7 Winn, B., Whitaker, D., Elliott, D. B. & Phillips, N. J. Factors affecting light-adapted pupil size in normal human subjects. *Investigative ophthalmology & visual science* **35**, 1132-1137 (1994).

8 Pandit, R. J. & Taylor, R. Mydriasis and glaucoma: exploding the myth. A systematic review. *Diabetic medicine : a journal of the British Diabetic Association* **17**, 693-699 (2000).

9 Wolfs, R. C., Grobbee, D. E., Hofman, A. & de Jong, P. T. Risk of acute angle-closure glaucoma after diagnostic mydriasis in nonselected subjects: the Rotterdam Study. *Investigative ophthalmology & visual science* **38**, 2683-2687 (1997).

10 Olden, M., Holle, R., Heid, I. M. & Stark, K. IDGenerator: unique identifier generator for epidemiologic or clinical studies. *BMC medical research methodology* **16**, 120 (2016).

11 Ferris, F. L., 3rd *et al.* Clinical classification of age-related macular degeneration. *Ophthalmology* **120**, 844-851 (2013).

12 Ferris, F. L. *et al.* A simplified severity scale for age-related macular degeneration: AREDS Report No. 18. *Archives of ophthalmology* **123**, 1570-1574 (2005).

13 Klein, R. *et al.* Harmonizing the classification of age-related macular degeneration in the three-continent AMD consortium. *Ophthalmic epidemiology* **21**, 14-23 (2014).

14 Kuha J, S. C. Categorical data analysis and misclassification. *In Survey Measurement and Process Quality* **1**, 633-670 (1997).

15 Kuha J., S. C., Palmgren J. *Misclassification error.*, (P Armitage and Th. Colton (eds) Wiley, 2005).

16 Tenenbein, A. A double sampling scheme for estimating from misclassified multinomial data with applications to sampling inspection. *Technometrics* **14**, 187-202 (1972).

17 Klaver, C. C. *et al.* Incidence and progression rates of age-related maculopathy: the Rotterdam Study. *Investigative ophthalmology & visual science* **42**, 2237-2241 (2001).

18 Korb, C. A. *et al.* Prevalence of age-related macular degeneration in a large European cohort: results from the population-based Gutenberg Health Study. *Graefe's archive for clinical and experimental ophthalmology = Albrecht von Graefes Archiv fur klinische und experimentelle Ophthalmologie* **252**, 1403-1411 (2014).

19 Davis, M. D. *et al.* The Age-Related Eye Disease Study severity scale for age-related macular degeneration: AREDS Report No. 17. *Archives of ophthalmology* **123**, 1484-1498 (2005).

20 Klein, R. *et al.* The Wisconsin age-related maculopathy grading system. *Ophthalmology* **98**, 1128-1134 (1991).

21 Jonasson, F. *et al.* Prevalence of age-related macular degeneration in old persons: Age, Gene/environment Susceptibility Reykjavik Study. *Ophthalmology* **118**, 825-830 (2011).

22 Harris, T. B. *et al.* Age, Gene/Environment Susceptibility-Reykjavik Study: multidisciplinary applied phenomics. *American journal of epidemiology* **165**, 1076-1087 (2007).

23 Augood, C. A. *et al.* Prevalence of age-related maculopathy in older Europeans: the European Eye Study (EUREYE). *Arch.Ophthalmol.* **124**, 529-535 (2006).

24 Vingerling, J. R. *et al.* The prevalence of age-related maculopathy in the Rotterdam Study. *Ophthalmology* **102**, 205-210 (1995).

25 Hofman, A. *et al.* The Rotterdam Study: 2012 objectives and design update. *European journal of epidemiology* **26**, 657-686 (2011).

26 Mitchell, P., Smith, W., Attebo, K. & Wang, J. J. Prevalence of age-related maculopathy in Australia. The Blue Mountains Eye Study. *Ophthalmology* **102**, 1450-1460 (1995).

27 Wilde, C. *et al.* Prevalence of age-related macular degeneration in an elderly UK Caucasian population-The Bridlington Eye Assessment Project: a cross-sectional study. *Eye*, https://doi.org/10.1038/eye.2017.30 (2017).

28 Bird, A. C. *et al.* An international classification and grading system for age-related maculopathy and age-related macular degeneration. The International ARM Epidemiological Study Group. *Survey of ophthalmology* **39**, 367-374 (1995).

29 Akuffo, K. O. *et al.* Prevalence of age-related macular degeneration in the Republic of Ireland. *The British journal of ophthalmology* **99**, 1037-1044 (2015).

30 Klein, R., Klein, B. E. & Linton, K. L. Prevalence of age-related maculopathy. The Beaver Dam Eye Study. *Ophthalmology* **99**, 933-943 (1992).
